# Longer-Term Follow-Up of Kenyan Men Circumcised Using the ShangRing Device

**DOI:** 10.1371/journal.pone.0137510

**Published:** 2015-09-14

**Authors:** Paul J. Feldblum, Jairus Okech, Rolex Ochieng, Catherine Hart, Grace Kiyuka, Jaim Jou Lai, Valentine Veena

**Affiliations:** 1 FHI 360, Durham, North Carolina, United States of America; 2 Homa Bay District Hospital, Homa Bay, Kenya; 3 FHI 360, Nairobi, Kenya; Cardiff University, UNITED KINGDOM

## Abstract

**Objectives:**

To ascertain clinical sequelae, client satisfaction and sexual behavior 2+ years after male circumcision using the ShangRing device.

**Methods:**

We enrolled 199 men from the Kenya sites (Homa Bay district) participating in a 2012 study of the ShangRing device used in routine male circumcision services (N = 552). We enrolled men who had had the ShangRing placed successfully, and over-sampled men who had had an adverse event and/or were HIV-positive during the field study. In the present study, each participant was examined and interviewed by a study clinician, and penile photographs were taken to document longer-term cosmetic results and any abnormal findings.

**Results:**

194 men were included in the analysis. The mean and median times between circumcision and the longer-term follow-up visit in this study were 31.8 and 32 months, respectively. Four men (2.1%) had signs/symptoms of a sexually transmitted infection (STI). Virtually all (99.5%) of the men were very satisfied with the appearance of their circumcised penis, and all would recommend a ShangRing circumcision to friends or family members. The most prevalent reported advantage of the circumcision was the ease of bathing and enhanced cleanliness of the penis (75.8%). 94.3% of the men did not cite a single negative feature of their circumcision. 87.5% of men reported more sexual pleasure post-MC, the most common reason being more prolonged intercourse. The majority of men (52.6%) reported one sexual partner post-MC, but more than a quarter of the men (28.1%) reported an increased number of partners post-MC. Less than half of the men (44.3%) reported using condoms half of the time or more, but the great majority of condom users stated that condom use was much easier post-MC, and 76.9% of users said they used condoms more after circumcision than before.

**Conclusions:**

This study supports the safety and acceptability of ShangRing male circumcision during 2–3 years of follow-up. It should allay worries that the ShangRing procedure could lead to delayed complications later than the observation period of most clinical studies.

**Trial Registration:**

ClinicalTrials.gov NCT01567436

## Introduction

Multiple randomized trials have shown that adult voluntary medical male circumcision (VMMC) provides enduring protection against HIV acquisition among men [1–3]. The World Health Organization (WHO) and the Joint United Nations Programme on HIV/AIDS (UNAIDS) recommended that voluntary medical male circumcision (VMMC) should be considered an effective tool for HIV prevention [4].

As VMMC is scaled up, the safety, effectiveness, efficiency and acceptability of procedures becomes more important. Male circumcision (MC) devices have the potential to accelerate the pace of VMMC programs [5], and two such devices have been pre-qualified by the WHO for routine use [6–7], including the ShangRing, manufactured in China [7]. Wound healing after conventional surgical MC is by primary intention, meaning suturing is done for wound closure, and the great majority of surgically circumcised men are completely healed within six weeks [8]. But healing following ShangRing circumcision procedures is by secondary intention, meaning the wound is allowed to close naturally without suturing, which tends to take longer than after surgical procedures [8].

Since study participants in male circumcision research are typically followed only through approximately six weeks post-procedure, longer-term adverse consequences of device circumcision that might result from the slower secondary intention healing process have received little attention. The cosmetic appearance of the penis after ShangRing circumcision also warrants attention. Longer-term follow-up data would inform recommendations on wide-scale introduction of devices [8]. We conducted a survey of Kenyan men who had undergone circumcision using the ShangRing device more than two years prior as part of an earlier study. The present follow-up survey assesses potential longer-term adverse consequences such as unsatisfactory cosmetic results or diminished sexual function.

## Methods

A large prospective study of the ShangRing was conducted in Kenya and Zambia in 2012; that field study is described in detail elsewhere [9]. Briefly, the Kenyan portion (N = 552) was done in routine service delivery settings at seven sites in Homa Bay District, Nyanza Province, Western Kenya, coordinated by the Homa Bay District Hospital. The primary objective of the field study was to measure the frequency of adverse events during the circumcision procedure and throughout follow-up until complete wound healing. Healthy, uncircumcised men ages 18–54 years old seeking circumcision were informed of the study. Men with active genital infection, or an anatomic abnormality or condition that contraindicated elective surgery under local anesthesia (e.g. significant systemic illness) were ineligible. Interested men underwent HIV prevention and risk-reduction counseling per national guidelines. Both HIV-negative and HIV-positive men were enrolled. Following administration of written informed consent and clinical screening procedures, the circumcisions were performed by providers with extensive surgical MC experience. Providers’ experience with the ShangRing varied from recent trainees to some who were considered master trainers on the device. Details of the ShangRing placement and removal techniques can be found elsewhere [10]. Follow-up visits were scheduled at Day 7 after circumcision for device removal, and at Day 35–42 after circumcision to assess wound healing.

The primary objective of the follow-up study described in this report was to identify longer-term (2+ years post-circumcision) sequelae using the ShangRing device. We also sought to evaluate overall client satisfaction with the procedure, self-reported post-circumcision changes in sexual pleasure, condom use, and numbers of sexual partners, and attitudes and satisfaction of the men’s main sexual partner, as reported by the male participants.

We aimed to enroll 200 men from in and around Homa Bay town, using the locater information in the stored study files from the field study. (Participants in the earlier study had consented to be contacted about future research studies.) To be eligible for the follow-up study, men had to: be a participant in the field study who received a ShangRing circumcision; be willing to have penile photographs taken; and be willing to undergo the informed consent process, sign the consent form and provide locater information including cell phone number. We intended to enroll all 11 men in the field study who had had a moderate/severe adverse event (AE), and at least 20 participants who were HIV-positive at the field study screening. Each participant made a single visit, during which he was examined and interviewed by an experienced study clinician (JO, RO or GK), all of whom worked in the initial field study. Penile photographs were taken to document longer-term cosmetic results and any abnormal findings. Visits took place at one of the field study clinics or (with consent) at the men’s homes.

Ethical committee review and approval for both the field study and this longer-term follow-up of men were obtained from both the FHI 360 and the Kenya Medical Research Institute IRBs.

## Results

### Baseline features of the follow-up cohort

We located, consented, enrolled, interviewed and examined 199 men from September 2014-January 2015. The identification numbers of three men could not be matched to participants from the prior field study; for two other men it could not be confirmed that circumcision was done using the ShangRing. These five men were excluded, leaving 194 men in the analysis data set (Table 1). At the time of the circumcision procedures, their mean and median ages were 24.8 and 22 years, respectively (interquartile range [IQR] 18–29). About 80% had had sexual intercourse in the 12 months prior to MC, with 71.0% of those men reporting zero or one sexual partner in the past 6 months and 40.6% reporting consistent condom use (Table 1).

**Table 1:** Selected Baseline Features of Men at ShangRing Circumcision Visit who were Interviewed 2+ Years Later (N = 194).

Baseline Factors	N	%
**Age in years**		
18–20	90	46.4
21–24	29	14.9
25–30	34	17.5
31+	41	21.1
Mean (± s.d.)	24.8	(± 8.0)
Median (interquartile range)	22	(18–29)
**Had sexual intercourse in past year**		
No	39	20.1
Yes	155	79.9
**Number of sexual partners in last 6 months** *		
0	6	3.9
1	104	67.1
2	30	19.4
3 or more	15	9.6
**Current condom use frequency** *		
Never use	44	28.4
Use less than half the time	17	11.0
Use about half the time	15	9.7
Use more than half the time	16	10.3
Always use	63	40.6

* Among 155 men reporting intercourse in past year.

The mean and median time between date of circumcision and date of follow-up were 31.8 and 32 months, respectively (interquartile range [IQR] 31–33 months).

### Results of genital examinations and medical history

Four men (2.1%) had signs/symptoms of a sexually transmitted infection (STI) at follow-up, and one man (0.5%) reported difficulty with erections since circumcision. No participant reported penile torsion, urinary problems, or any other complication or abnormality.

### Acceptability parameters

**Fig 1:**
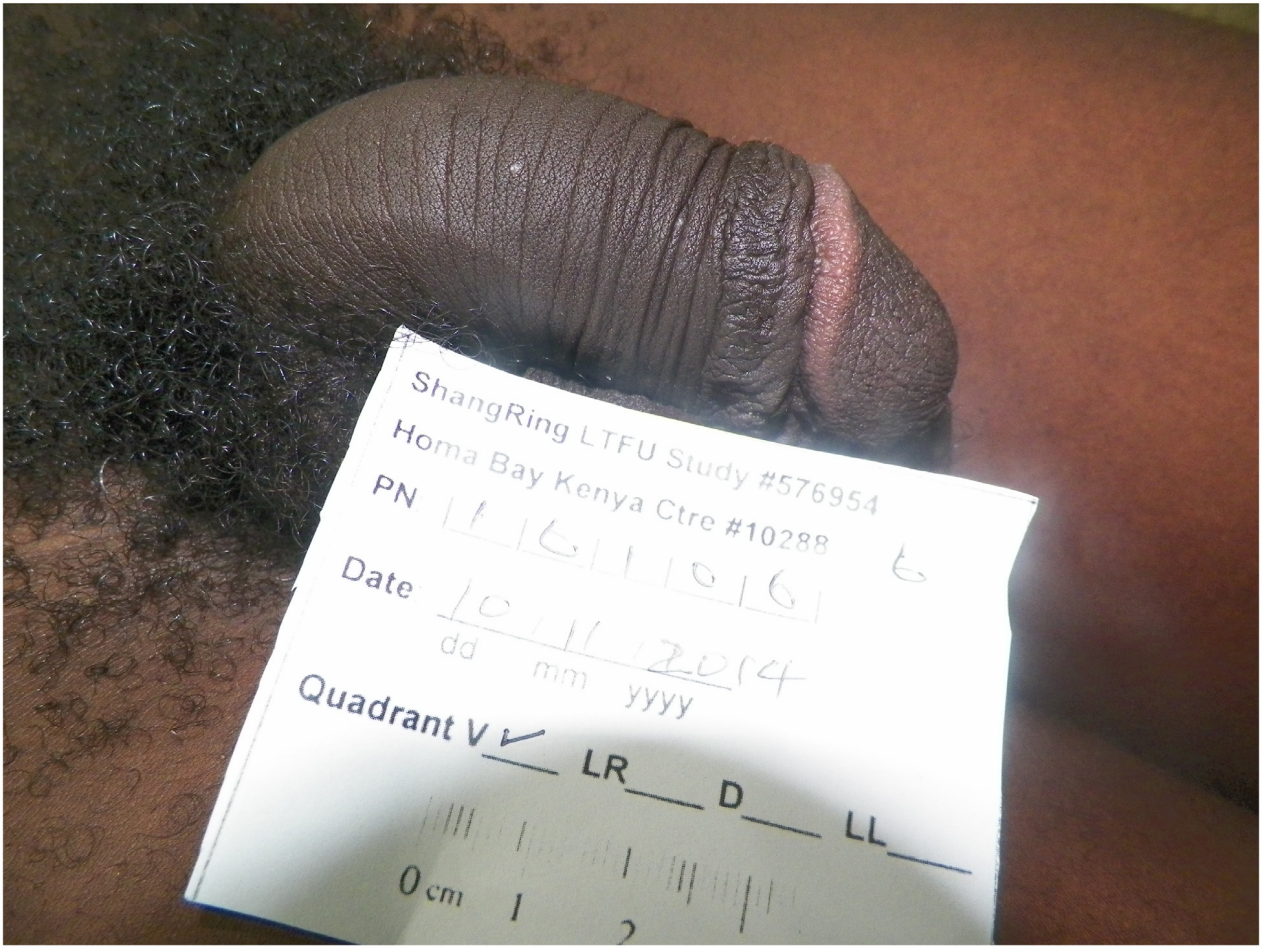
Typical Penile Appearance at Longer-term Follow-up.

**Fig 2:**
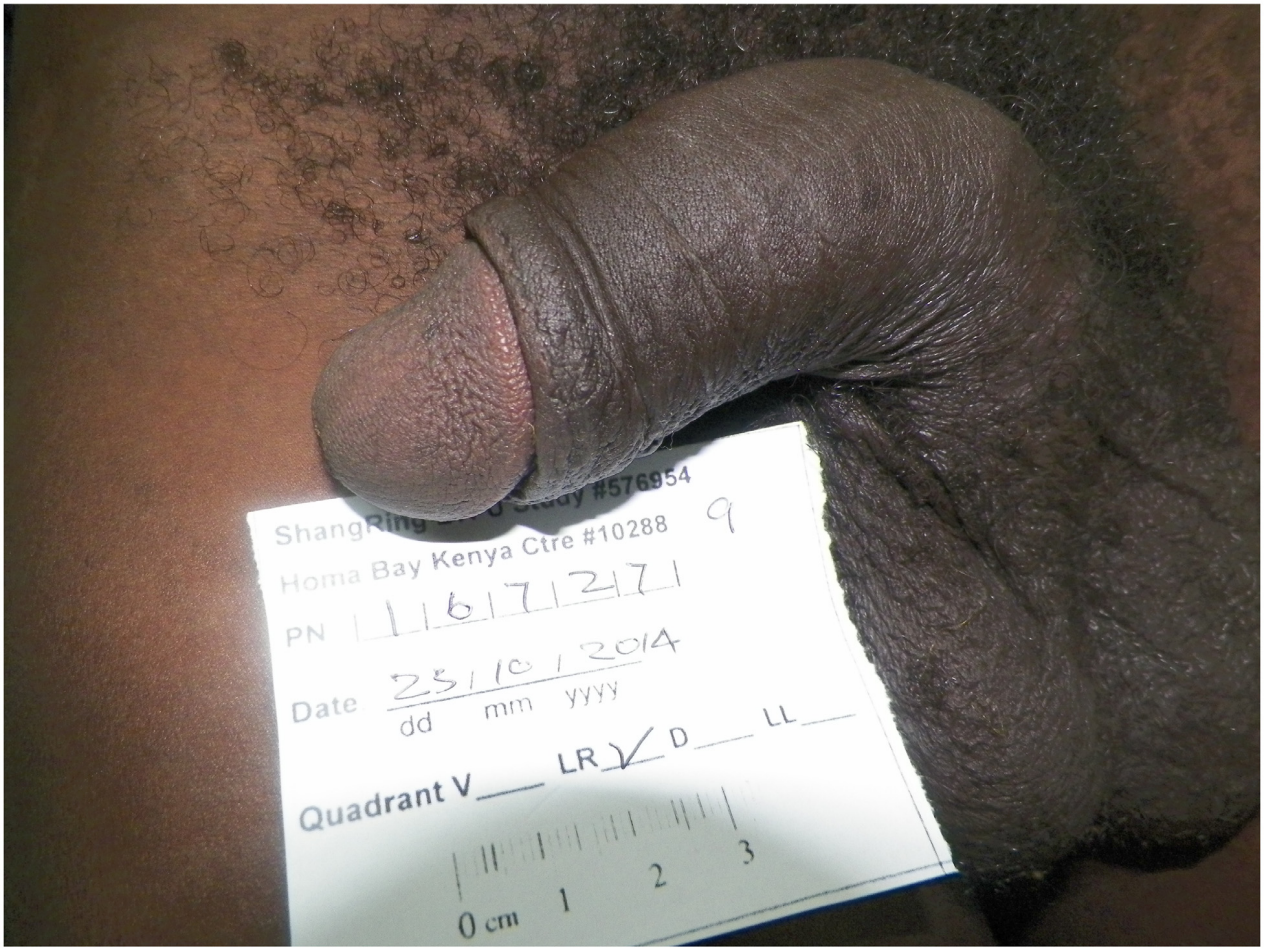
Typical Penile Appearance at Longer-term Follow-up.

Virtually all (193, 99.5%) of the men stated they were very satisfied with the appearance of their circumcised penis (see Figs 1 and 2 for typical penile appearance), and all participants said they would recommend a ShangRing circumcision to friends or family members. The most common specific reason given for their willingness to recommend the ShangRing procedure was that there was less pain than expected (48.2%), followed by the rapid healing process (14.5%) and the cosmetic appearance (13.0%). The most common response to the question “What is the one best thing about your circumcision?” was the ease of bathing and the enhanced cleanliness of the penis (75.8%). 94.3% of the men did not cite a single negative feature of their circumcision; 4.2% mentioned mild pain as the worst feature they recalled.

When asked how their sexual pleasure had been affected by circumcision, 87.5% of men reported more pleasure post-MC, 1.0% reported less pleasure, and 11.5% said there was no change or they did not know. Among those men who reported more pleasure, the most common reason given was more prolonged intercourse (57.4%), followed by easier penetration (16.6%) and freedom from penile laceration or pain (16.6%). We asked men to opine about their main sexual partner’s pleasure during intercourse. One hundred fifty-five men (80.3%) reported more pleasure for their partner, one man (0.5%) reported less pleasure, and 19.2% reported no change or were not sure about partner’s pleasure. The most common specific reason given for partners’ increased pleasure was more prolonged intercourse (32.5%).

### Sexual behavior

5.2% of men reported no sexual partners in the past six months, while the majority of men (52.6%) reported one sexual partner, 26.3% reported two partners, and 16.0% reported three or more partners (Table 2). Those figures represented no change for most of the men, but more than a quarter (28.1%) responded that they had an increased number of partners post-MC (Table 2).

**Table 2:** Selected Sexual Behavior Features of Men Interviewed 2+ Years after ShangRing Male Circumcision (N = 194).

Post-MC Behavioral Factors	N	%
**No. of sexual partners in last six months (N = 194)**		
0	10	5.2
1	102	52.6
2	51	26.3
3 or more	31	16.0
**Change in no. of sexual partners after male circumcision (N = 192)** *		
No change	122	63.5
Increased	54	28.1
Decreased	16	8.3
**Frequency of condom use after male circumcision (N = 194)**		
Never	47	24.2
Sometimes	61	31.4
Half of the time	3	1.6
Most of the time	36	18.6
Every time	47	24.2
**Change in condom use frequency after male circumcision (N = 147)** *		
No change	26	17.7
Use condoms more	113	76.9
Use condoms less	5	3.4
Don’t know / no response	3	2.0
**Ease of condom use after male circumcision (N = 144)**		
Much easier	136	94.4
Easier	6	4.2
About the same	1	0.7
More difficult	1	0.7

* Based on recall of the respondents at the time of longer-term follow-up.

Less than half of the men (44.3%) reported using condoms half of the time or more (Table 2). But the great majority (94.4%) of users stated that condom use was much easier post-MC, and 76.9% of users said they used condoms more after circumcision than before.

## Discussion

Our study follows others that have examined the impacts of adult male circumcision on health and sexual function. For example, a large two-year follow-up of men with conventional surgical circumcision found no evidence of penile deformities or complications [11]. Male circumcision has not been shown to result in adverse changes in sexual function or satisfaction [12]. But given the relative novelty of devices for MC, longer-term follow-up data are scanty. Yue and colleagues [13] conducted a study of sexual function among 98 men circumcised using the ShangRing a mean of 19 months earlier (range 9 to 28 months). Compared with pre-circumcision interviews, they found mainly slight and non-significant differences in sexual function and satisfaction, though sexual frequency and partner satisfaction were reported to be significantly higher in the post-circumcision interviews. No important complications were observed, and the median satisfaction score for the ShangRing was 8 on a 10-point scale.

Similarly, our analysis aimed to elucidate complications of ShangRing circumcisions occurring after the observation period of typical clinical studies. We also sought to ascertain levels of longer-term acceptability, and impacts on sexual pleasure and behavior. Only a few men had evidence of any genito-urinary problems, most of which were apparent STIs. Acceptability of and satisfaction with ShangRing male circumcision were nearly universal. The majority of men reported greater sexual pleasure for themselves and their main partners following ShangRing circumcision.

Comparing participants’ responses regarding sexual behavior at this longer-term follow-up to their responses at the time of the ShangRing circumcision yielded hints of increased sexual risks among these Kenyan men. In the earlier study, 26.3% of the Kenyan participants reported two or more sex partners in the past six months; 60.6% reported using condoms about half the time or more, including 40.6% who reported always using condoms. 42.3% of men in the follow-up study reported two or more partners; 44.3% of the men reported using condoms half of the time or more post-MC, including 24.2% who reported using condoms all the time. These figures are difficult to reconcile with the 77% of responders in the follow-up study who stated that their condom use had increased after circumcision. The veracity of self-reported condom use in reproductive health studies is problematic.

Our longer-term follow-up study enrolled approximately one-third of the men who participated in the previous ShangRing field study at the Kenya site [9]. Baseline features of men included in this longer-term follow-up were similar to those of men not included in the study, although the average baseline age of men followed was about 2 years older than men not followed-up. We intentionally over-sampled field study participants who were HIV-infected, and/or who experienced an adverse event, a conservative approach that might yield higher rates of complications detected at follow-up. Thus 37 of 194 men followed-up here (19.1%) were HIV-infected and/or had an adverse event in the field study, a higher percentage than were found to be HIV-infected in the prior field study (8.7%). Even so, the rates of various complications at follow-up were quite low. Given the relatively small number of follow-up study men with HIV infection and/or an AE in the field study, we did not compare or present the complication rates according to prior HIV status/AE occurrence.

A weakness of our follow-up study is that we had limited data on pre-circumcision sexual behavior and none on sexual functioning, unlike the Yue study [13]. We also lacked a quantitative scale to index sexual function. While we determined HIV infection status in the prior field study, we did not perform HIV testing in the follow-up study and so could not identify men who might have seroconverted during the intervening months. Finally, although it would be important to compare results between men with and without an AE in the prior field study, and between HIV-negative and HIV-infected men, our study size was too small to permit meaningful comparisons.

A recent systematic review reveals that short-term endpoints of ShangRing circumcision compare favorably with conventional surgical procedures [14]. Despite the limitations noted above, the current study sheds light on the safety and acceptability of ShangRing male circumcision 2–3 years after the procedure. Our study and that of Yue et al [13] should allay worries that the ShangRing procedure might lead to delayed complications after the observation period of most clinical studies.

## Supporting Information

S1 Protocol Trial Protocol (PDF)

S1 TREND Checklist TREND Checklist. (PDF)
