# Supplementary material for: Longer-Term Follow-Up of Kenyan Men Circumcised Using the ShangRing Device
Source: PLoS One. 2015 Sep 14;10(9):e0137510. doi: 10.1371/journal.pone.0137510 (PMC4569077; doi:10.1371/journal.pone.0137510)
Supplement: S1 Protocol — (PDF) [file pone.0137510.s001.pdf]

# Longer-term Follow-up of Men Who Were Circumcised Using the ShangRing Device in Kenya

## FHI 360 PHSC Study #576954 / KEMRI Non-SSC Protocol #446


**Sponsor:** **FHI 360**
359 Blackwell Street, Suite 200
Durham, NC 27701 USA



**Funder:** **Bill & Melinda Gates Foundation**



**Principal Investigator:** **Paul Feldblum, PhD, Scientist II**
FHI 360
359 Blackwell Street, Suite 200
Durham, NC 27701 USA
Telephone: +1-919-544-7040 ext. 11237
Fax: +1-919-544-7261
E-mail: pfeldblum@fhi360.org

**FHI 360 Project Manager:** **Catherine Hart, AAS, CCRP, Research Associate II**
FHI 360
359 Blackwell Street, Suite 200
Durham, NC 27701 USA
Telephone: +1-919-544-7040 ext. 11367
Fax: +1-919-544-7261
E-mail: chart@fhi360.org

**Site Investigator:** **Jairus Okumu Oketch**
Homa Bay District Hospital
P.O BOX 52 HOMA-BAY
Mobile: 254 724 579 750 / 254 07337649596
E-mail: jairusoketch@yahoo.com

# Longer-term Follow up of Men Circumcised with ShangRing in Kenya

## FHI 360 Study #546954 / KEMRI Non-SSC Protocol #446

## SITE INVESTIGATOR'S STATEMENT

I, **Jairus Oketch**, the Site Investigator, agree to conduct this study in full accordance with the provisions of this protocol and will comply with all requirements regarding the obligations of clinical investigators as fully outlined in the International Conference on Harmonization (Section E6(R1) Good Clinical Practice), local regulatory requirements, and in the FHI 360 Statement of Investigator which I have also signed. I agree to maintain all study documentation until FHI 360 advises that it is no longer necessary. I also agree to publish or present data only upon review and after discussion with FHI 360.

I have read and understand the information in this protocol, including the potential risks and side effects of the product under investigation, and will ensure that all associates, colleagues, and employees assisting in the conduct of the study are informed about the obligations incurred by their contribution to the study.

______________________________ ______________________________

**Signature of Site Investigator** **Date (dd/mm/yyyy)**

______________________________ ______________________________

**Signature of FHI 360 Project Leader** **Date (dd/mm/yyyy)**

Prepared by:
Clinical Sciences Unit
FHI 360
359 Blackwell Street, Suite 200
Durham, NC 27701 USA
Tel: 1-919-544-7040
Fax: 1-919-544-7261

# ACRONYMS

| | |
|---|---|
| **AE** | Adverse Event |
| **CFR** | U.S. Code of Federal Regulations |
| **CRF** | Case Report Form |
| **GCP** | Good Clinical Practice |
| **HIV** | Human Immunodeficiency Virus |
| **ICF** | Informed Consent Form |
| **IRB** | Institutional Review Board |
| **KEMRI** | Kenya Medical Research Institute |
| **MDDF** | Manual Data Discrepancy Form |
| **MMC** | Medical Male Circumcision |
| **M&E** | Monitoring and Evaluation |
| **MOH** | Ministry of Health |
| **PI** | Principal Investigator |
| **PHSC** | Protection of Human Subjects Committee (FHI 360) |
| **SI** | Site Investigator |
| **SOP** | Standard Operating Procedure |
| **STI** | Sexually Transmitted Infection |
| **UNAIDS** | Joint UN Programme on HIV/AIDS |
| **USAID** | U.S. Agency for International Development |
| **VMMC** | Voluntary Medical Male Circumcision |
| **WHO** | World Health Organization |

# Table of Contents

**ACRONYMS** .......... **3**

**STUDY SUMMARY** .......... **6**

**1.0 INTRODUCTION** .......... **7**

1.1 Background .......... 7

1.2 Rationale .......... 7

**2.0 OBJECTIVES AND ENDPOINTS** .......... **8**

2.1 Objectives .......... 8

*2.1.1 Primary Objective* .......... 8

*2.1.2 Secondary Objectives* .......... 8

2.2 Endpoints .......... 8

*2.2.1 Primary Endpoint* .......... 8

*2.2.2 Secondary Endpoints* .......... 8

**3.0 STUDY PARTICIPANTS** .......... **8**

3.1 Study Participant Selection .......... 9

3.2 Inclusion Criteria .......... 9

3.3 Exclusion Criteria .......... 9

**4.0 RESEARCH STUDY DESIGN** .......... **9**

4.1 Study Visits .......... 9

4.2 Recruitment .......... 10

4.3 Study Sites and Duration .......... 10

**5.0 PROTECTION OF HUMAN SUBJECTS** .......... **11**

5.1 Institutional Review .......... 11

5.2 Informed Consent .......... 11

5.3 Risks and Benefits .......... 11

5.4 Participant Confidentiality .......... 12

5.5 Compensation .......... 12

**6.0 DATA MANAGEMENT** .......... **12**

6.1 Overview .......... 12

6.2 Record Retention and Data Storage .......... 12

6.3 Data Collection .......... 13
6.4 Data Entry .......... 13
**7.0 DATA ANALYSIS .......... 13**
**8.0 PUBLICATION AND PRESENTATION OF RESULTS .......... 14**
**REFERENCES .......... 15**

# STUDY SUMMARY

| | |
|---|---|
| **Design** | An acceptability and safety assessment of men who were circumcised using the ShangRing device in Kenya. |
| **Population:** | 200 men from Kenya who were previously circumcised using the ShangRing device as participants in the study entitled, "A Prospective Observational Study of Male Circumcision Using the ShangRing in Routine Clinical Settings in Kenya & Zambia," with special emphasis on men who were HIV-positive when they underwent circumcision, or who had experienced an adverse event during the study. |
| **Duration:** | Total expected duration of approximately 4 months in the field. |
| **Primary Objective:** | Identify longer-term (1+ years) sequelae and client satisfaction with adult medical male circumcision using the ShangRing device. |
| **Primary Endpoint:** | Prevalence of complications detected at examination such as excessive scarring/keloid formation and torsion; self-reported sequelae such as erectile problems or urination difficulties; and self-reported satisfaction with the ShangRing procedure and cosmetic appearance, by standardized interview. |
| **Secondary Objectives:** | • To assess female partner's attitudes and satisfaction with the procedure, as reported by the male participant<br>• To evaluate self-reported post-procedure changes in sexual pleasure, condom use, and numbers of sexual partners. |
| **Secondary Endpoints:** | We will calculate the percentages of men reporting specific items on the standardized interview questionnaire. |
| **Site:** | Homa Bay District Hospital, Nyanza Province, Kenya |

# 1.0 INTRODUCTION

## 1.1 Background

Multiple randomized trials have shown that adult medical male circumcision (MMC) prevents HIV acquisition among men in sustained fashion (Gray et al. 2012; Mehta et al. 2013; Auvert et al. 2013). In addition to preventing HIV infection, studies have also shown MMC to prevent HPV and HSV infections (Zetola et al. 2012). The World Health Organization (WHO) and the Joint United Nations Programme on HIV/AIDS (UNAIDS) recommend that medical male circumcision (MMC) should be considered an effective tool for HIV prevention (WHO 2007).

As MMC is scaled up, the long-term safety and acceptability of procedures becomes more important. The use of the ShangRing and other devices for MMC results in healing by secondary intention once the device is removed. While several studies have documented complete healing and acceptability of circumcision using the ShangRing method in Kenya (Barone et al. 2011, Barone et al. 2012, Sokal et al. 2014), men were followed only through approximately six weeks post-circumcision. It is unknown whether there are any longer-term adverse consequences of adult circumcision healing by secondary intention, as occurs with the ShangRing. These might include untoward scar formation late in the healing process and changes/decrements in sexual function.

## 1.2 Rationale

Per the WHO's most recent summary guidance on the use of devices for adult MMC, additional data on longer-term outcomes would provide valuable information to help the WHO make recommendations about wide-scale introduction of devices (WHO 2012). Therefore, the WHO advised that it would be prudent to conduct a survey of men at a minimum of one year post-circumcision to assess potential longer-term adverse consequences such as keloid formation, as well as potential effects on sexual function and final cosmetic results.

For this research study, we will contact and gather data on at least 200 men who participated in the earlier ShangRing study entitled, "A Prospective Observational Study of Male Circumcision Using the ShangRing in Routine Clinical Settings in Kenya & Zambia" (PHSC reference #10278 / KEMRI reference # Non-SSC Protocol 229). We will not recruit or include men who were circumcised using the ShangRing during the earlier randomized controlled trial of the ShangRing or other ShangRing studies conducted in the same area in western Kenya.

# 2.0 OBJECTIVES AND ENDPOINTS

## 2.1 Objectives

### *2.1.1 Primary Objective*

The primary objective of this research study is to identify longer-term (1+ years post-procedure) sequelae and overall client satisfaction with adult MMC using the ShangRing device, especially among men who were HIV-positive at the time of ShangRing circumcision and men who had an adverse event during the earlier prospective observational ShangRing study.

### *2.1.2 Secondary Objectives*

The secondary objectives of this research study are:

- To evaluate self-reported changes in sexual pleasure, condom use, and numbers of sexual partners.
- To assess main female partner's attitudes and satisfaction with the procedure, as reported by the male participant

## 2.2 Endpoints

### *2.2.1 Primary Endpoint*

The prevalence of complications such as excessive scarring/keloid formation and torsion; self-reported sequelae including erectile problems and urination difficulties; and satisfaction with the ShangRing procedure and cosmetic appearance. These will be ascertained using physical examination, penile photography, and a standardized interview.

### *2.2.2 Secondary Endpoints*

We will calculate the percentages of men responding affirmatively to items on the standardized questionnaire to address secondary objectives.

# 3.0 STUDY PARTICIPANTS

We will enroll at least 200 men primarily from in and around Homa Bay town who were previously circumcised using the ShangRing method during their participation in the study entitled "A Prospective Observational Study of Male Circumcision Using the ShangRing in Routine Clinical Settings in Kenya & Zambia." The study files with locator information are stored at the Homa Bay District Hospital and are accessible to the Site Investigator. The informed consent form (ICF) in the prior study included consent for contact to join future research studies.

## 3.1 Study Participant Selection

In selecting study participants, we begin enrollment with all 11 men who reported an MC-related adverse event (AE) during their participation during the prior prospective observational study. Next, we will attempt to enroll at least 20 men who were enrolled with an HIV-positive status during the prior study, regardless of location. We will otherwise focus enrollment efforts among residents in Homa Bay town for efficiency reasons. If we fall short of the overall enrollment target of 200 men currently living in Homa Bay town, we will widen recruitment to the more peripheral areas of Ndiru, Nyagoro, and Gongo where the prospective observational study was conducted.

For the men with AEs and/or HIV-infected status in the prior study, we will make three attempts to contact each of them and invite them to enroll in this study. For the other residents of Homa Bay town (and Ndiru, Nyagoro, and Gongo if necessary), site staff will make one contact attempt each until 200 participants are enrolled.

## 3.2 Inclusion Criteria

In order to participate in this research study, men must meet the following inclusion criteria:

- Prior participation and circumcision with the ShangRing device during the study entitled "A Prospective Observational Study of Male Circumcision Using the ShangRing in Routine Clinical Settings in Kenya & Zambia";
- Willing to have penile photographs taken;
- Must freely consent to participate in the study, sign an informed consent form (ICF), and provide locator information; and,
- Must have a cell phone or access to a cell phone.

## 3.3 Exclusion Criteria

- Did not receive a circumcision using the ShangRing device during the prior prospective observational ShangRing study.

# 4.0 RESEARCH STUDY DESIGN

## 4.1 Study Visits

This study will consist of one visit per participant. Men will be examined by a study clinician (the Site Investigator or his designee) as well as interviewed. Penile photographs will be taken (with consent) to document long-term cosmetic results and any abnormal findings. The visits can take place at the clinic or (with consent) at the men's homes.

## 4.2 Recruitment

Study staff will contact men by telephone to assess their interest in learning more about the study. A telephone script will be used for the calls, which will provide staff with language to use if contact is made with the man including requesting the man's birth date as a way to confirm the man's identity). Men will be told the reason for the phone call, asked if it is okay to speak with them, and briefly introduced to the study. Men will then be asked if they are interested in making an appointment to learn more about the study. Men will not be consented over the telephone. Complete informed consent will be conducted when men present for their appointments and before any study procedures, as stated in Section 5.2 below. Men who decline to make the appointment will be thanked for their time and not be contacted again. If a reason for declining to make the appointment is offered by the man, we will make note of the reason on the telephone log, but men will not be required to give us a reason. Only the man's unique participant number from the prior prospective observational ShangRing study will be used on the telephone log. Names will not be used on the telephone log.

If the study staff person has to leave a phone message, a script will also be used for leaving a message. The message will only contain the staff person's name and phone number with the request to return the call. No information about the current study or the man's previous participation in the former ShangRing study will be used in the message to ensure confidentiality.

During study specific training, staff will be trained on using the script and phone recruitment procedures.

## 4.3 Study Sites and Duration

This study will be conducted at the Homa Bay District Hospital, Nyanza Province, Kenya.

Study staff will be thoroughly trained in all study procedures, completion of data collection forms, additional study tools, phone recruitment, research ethics, and good clinical practice prior to study start.

Study duration is estimated to be approximately 4 months in the field. Additional time will be needed to complete the protocol and study-related instruments, obtain ethical approvals, and conduct the data analysis and report writing. Total time expected is approximately 9 months from start to finish.

# 5.0 PROTECTION OF HUMAN SUBJECTS

## 5.1 Institutional Review

This research study will not be implemented without documented approval by the local Kenyan institutional review board (IRB) and by FHI 360's Protection of Human Subjects Committee (PHSC). The Site Investigator must provide FHI 360 with written documentation, signed by the IRB Chairperson(s) or designee, stating that the KEMRI has approved the research protocol and the informed consent form (ICF).

The Site Investigator will be responsible for submitting progress and a final report to the local IRB (that of KEMRI) per its guidelines. These reports should include the total number of participants completing the study, all changes in research activity, as well as all unanticipated problems involving risk to study participants. All correspondence with KEMRI regarding this research study must be retained in the site's regulatory files and copies sent to FHI 360 for similar record keeping. The FHI 360 North Carolina study staff will be responsible for making all reports and submissions to the PHSC.

## 5.2 Informed Consent

A man will not be enrolled into this research study until the Site Investigator has obtained his legally effective (signed and/or witnessed), freely given, written informed consent (IC). The Site Investigator shall seek such consent under circumstances that provide the prospective participant with sufficient opportunity to learn about the study and consider whether or not to participate in it. IC will be obtained without coercion, undue influence or misrepresentation of the potential benefits and risks that might be associated with participation in the research study.

## 5.3 Risks and Benefits

There is no direct benefit for being interviewed and undergoing a physical examination. If a physical problem is noted during the examination, however, the man will be referred for treatment.

There is a risk of a breach in the confidentiality of information collected. As specified in Section 5.4 below, various steps will be taken to ensure the confidentiality of respondents and security of data. These procedures will be regularly monitored during fieldwork. There is also a slight risk that participants may feel uncomfortable talking about sensitive sexual behavior issues. Further, participants will be advised during the IC process that they may refuse to answer any question, or any part of a question, or discontinue the questioning at any time without consequence to their ability to receive treatment at the clinic.

## 5.4 Participant Confidentiality

The confidentiality of all participants enrolled in this research will be protected to the fullest extent. Site staff will receive training in research ethics with emphasis on the importance of maintaining confidentiality. All study paper records including ICFs, CRFs and locator forms will be kept in locked file cabinets with access limited to study staff only. Any electronic data records will not include participant identifying information and will be stored on password secured computers. Participants' information will be filed by participant ID number. Participant numbers will be entered on a master participant log that links names and client numbers with those participant numbers; that master log will be stored in the locked file cabinet with restricted access to study staff only. Photographs will only have participant numbers, never names, associated with them. They will be stored on password secured computers with access limited to designated study staff only.

Participants' research forms and research-related documentation may be audited by FHI 360 staff, regulatory personnel, or other individuals authorized to audit the research study in Kenya. However, participants will not be identified by name on any CRF, questionnaire, photograph, or any other research documentation sent outside the clinic. Participant identifiers, such as names and faces, will not be shown in or written on any photograph taken during this research study. All computerized and electronic database records will identify participants by identification numbers only. Participants will not be reported by name in any report or publication resulting from data collected in this study.

## 5.5 Compensation

We will give each participant 700 Kenyan Shillings (about $8.00 USD) to cover the cost of travel to/from the clinic for the interview and examination.

# 6.0 DATA MANAGEMENT

## 6.1 Overview

A detailed Data Management Plan will be written by FHI 360 prior to initiation of the study. In brief, data will be collected on a single 1-ply form; completed forms will be photocopied in the study office at the Homa Bay District Hospital. Original forms will be stored in the participant files at the hospital, while the photocopies will be sent to the FHI 360 North Carolina, USA office for data entry and storage.

## 6.2 Record Retention and Data Storage

All participant CRFs and any other source documents and Manual Data Discrepancy Forms (MDDFs), if applicable, will be securely stored at the Homa Bay District Hospital clinic in locked cabinets in separate folders by participant number located in a locked

clinic office with access only to study staff.  Only the Site Investigator and his study staff will have access to the forms.

When the study closes, all participant files will be transferred to the FHI 360 North Carolina office, and stored for a minimum of two years after study closure.

### 6.3 Data Collection

Data will be captured on 1-ply CRFs by the Site Investigator or his assistant through face-to-face interviews and physical examination of the participants.  The data collection visits can take place at the Homa Bay District Hospital or at the participant's home.

### 6.4 Data Entry

FHI 360 will be responsible for data entry and data management of the CRF data.  Data from the CRF will be entered into Epi Data™.  Data entry screens will be created; and, for each question on the CRF, the data entry screen will contain a field/variable name (name associated with the data/responses collected from each question on the CRF), description, type and code (selected value that can be used to populate other fields on form), if appropriate.

Data entry staff will inform the project manager of missing or inconsistent data.  It may be possible to make corrections by contacting the participants by telephone.  All CRFs that are sent to FHI 360 will be double entered.  The database will be backed up on a daily basis.  Data will then be exported from Epi Data into a SAS database.  All data-entry activities will follow FHI 360's SOP 06004: Data Collection Flow Process.

## 7.0 DATA ANALYSIS

A detailed analysis plan will be written and approved by FHI 360 prior to initiation of the study.  The plan will account for the inclusion of the men (maximum 11) with adverse events in the prior prospective observational study, and the over-sampling of HIV-positive men.  The characteristics of men in the prior study who did versus did not enroll in this study will be presented and compared so that we can uncover important selection factors.  All descriptive statistics will be presented separately by sampling status (had AE in prospective study, was HIV+ at time of circumcision, neither; with any HIV+ men who also had an AE contributing to both categories).

Data will be analyzed for safety and acceptability.  Safety analyses will include percentages of men with complications including excessive scarring/keloid and torsion, as well as self-reported issues such as erectile problems and urination difficulties.  We will tabulate participant responses regarding satisfaction with the ShangRing procedure and penile cosmetic appearance.  We will also tabulate self-reported changes in sexual

pleasure, condom use, and numbers of sexual partners, along with participants' perceptions of their main partners' attitudes and satisfaction with the procedure. The findings will be written and submitted for publication, and shared with the Kenyan IRB and the WHO Technical Advisory Group on male circumcision devices. The report will discuss the implications of the sampling method on generalizability of findings to ShangRing programs in general.

# 8.0 PUBLICATION AND PRESENTATION OF RESULTS

All publications emerging from the study will abide by FHI 360 Policy 05007: Authorship of Publications Reporting Data from FHI 360-sponsored Studies. As the study dataset will reside at FHI 360, FHI 360 staff will lead the main analysis. The publication of the main study results (primary study objective) will be shared by FHI 360 with the Kenyan Ministry of Health, as will analysis of secondary endpoints. Any presentations or publications on the study results will assign proper credit and acknowledgment to the contributions of FHI 360 and the MOH.

Presentations/publications on study secondary objectives, and secondary analyses of study data, may be undertaken by each partner who has the capability to utilize the data properly and professionally. Requests for data will be made to FHI 360 in writing and all collaborating partners will review all presentations or manuscripts for accuracy prior to submission. No data collected in this study may be published without prior written agreement of relevant staff from FHI 360 and the MOH.

All publications and final reports will be made available to the IRBs and regulatory agencies per their guidelines.

# REFERENCES

Auvert B, Taljaard D, Rech D, Lissouba P, Singh B, et al. Association of the ANRS-12126 Male Circumcision Project with HIV levels among men in a South African township: Evaluation of effectiveness using cross-sectional surveys. PLoS Med 2013; 10(9): e1001509.

Barone MA, Ndede F, Li PS, et al. The ShangRing device for adult male circumcision: a proof of concept study in Kenya. J Acquir Immune Defic Syndr 2011; 57(1): e7-12.

Barone MA, Awori QD, Li PS, et al. Randomized trial of the ShangRing for adult male circumcision with removal at one to three weeks: delayed removal leads to detachment. J Acquir Immune Defic Syndr 2012; 60(3): e82-9.

Gray R, Kigozi G, Kong X, et al. The effectiveness of male circumcision for HIV prevention and effects on risk behaviors in a posttrial follow-up study. AIDS 2012; 26(5):609-15.

Mehta SD, Moses S, Agot K, et al. Medical male circumcision and herpes simplex virus 2 acquisition: posttrial surveillance in Kisumu, Kenya. J Infect Dis. 2013; 208:1869-76.

Sokal DC, Li PS, Zulu R, et al. Randomized controlled trial of a minimally invasive circumcision device, the ShangRing, versus conventional surgical techniques for adult male circumcision: safety and acceptability. J Acquir Immune Defic Syndr 2014;65:447-55.

World Health Organization. Use of devices for adult male circumcision in public health HIV prevention programmes: executive summary. WHO: Geneva; 2012.

World Health Organization. Meeting Report: WHO Technical Advisory Group on Innovations in Male Circumcision: Evaluation of Two Adult Devices. WHO: Geneva; January 2013.

Zetola N, Klausner JD. Male Circumcision Reduces Human Papillomavirus Incidence and Prevalence: Clarifying the Evidence. Sex Transm Dis 2012; 39:114-5.
